# The Influence of Nordic Walking on Isokinetic Trunk Muscle Endurance and Sagittal Spinal Curvatures in Women after Breast Cancer Treatment: Age-Specific Indicators

**DOI:** 10.3390/ijerph18052409

**Published:** 2021-03-02

**Authors:** Justyna Hanuszkiewicz, Marek Woźniewski, Iwona Malicka

**Affiliations:** Faculty of Physiotherapy, University School of Physical Education in Wroclaw, 51-612 Wrocław, Poland; marek.wozniewski@awf.wroc.pl (M.W.); iwona.malicka@awf.wroc.pl (I.M.)

**Keywords:** Nordic walking, spine, trunk muscles, posture, aging, prevention, physical activity, breast cancer

## Abstract

Nordic walking (NW) is a popular form of rehabilitation. NW improves psychophysical condition in breast cancer (BC) survivors. This study aimed to analyze the effects of NW on functional and postural changes of the trunk in women of different ages after BC. We hypothesized that an age relationship would be found. BC survivors (*n* = 39) were stratified by age as “middle- aged” (45–59 years) or “older” (60–75 years), then randomly allocated to the training intervention. A study group (SG, *n* = 19) participated in NW and a control group (CG, *n* = 20) performed general gymnastics. The sagittal spinal curvatures and isokinetic trunk muscle endurance were recorded at two time points, pre- and post-training intervention. Significant within NW group changes (*p* < 0.05) were found for the total work (TW) and average power (AP) of trunk flexors and extensors and the upper thoracic angle in middle-aged women. In older Nordic walkers, significant increases in TW and AP of the trunk flexor muscles were observed, with a negative increase in the trunk inclination angle. In CG, no significant functional or postural changes were observed in response to general gymnastics. NW improved both functional and postural changes in middle-aged women. This study identified the limitations of NW training in older women.

## 1. Introduction

The aging process is natural and inevitable [1]. The typical changes associated with aging include deepening of the thoracic kyphosis, protrusion of the head, and in more severe cases, knee flexion. Balzini et al. reported that this flexed posture is related to nonosteoporotic conditions such as vertebral pain, emotional status and muscular impairment [2]. These factors are usually associated with decreased physical performance in older adults. An adequate level of physical activity (PA) plays a significant role in maintaining independent living and functional fitness. If older adults do not participate in a physically active lifestyle, they increase their risk of decreased muscle mass by 40%. In addition, a reduction in muscle mass of about 30% is associated with a loss of muscle strength. A 30% reduction in strength between 50 and 70 years of age has been demonstrated, followed by a more rapid decline over the next decades [1,3,4].

Moreover, older adults with chronic diseases show low muscle strength, which is strongly associated with poor health status and mortality, rather than low muscle mass [5]. Chronic diseases include breast cancer (BC), which is the most common cancer among women. The risk of developing BC increases with age, and the peak incidence is observed in postmenopausal women [6].

Adverse side effects of BC treatment such as restrictions on joint mobility, contractures, pain, muscle weakness, tissue fibrosis, peripheral nerve injuries, and disorders in the psychological sphere affect posture and the musculoskeletal system. Among BC survivors, 82.3% demonstrate a faulty posture (e.g., alterations in spine alignment, increased thoracic kyphosis and shoulder asymmetry). These changes affect the normal activity of the spine [7,8,9,10]. It was also demonstrated that back extensor strength decreases not only in response to aging, but also in response to reduced levels of PA [1,2]. Therefore, women after BC treatment need systematic and comprehensive rehabilitation to improve their physical condition. The standard form of BC rehabilitation in Poland is general gymnastics, which is aimed at improving upper extremity function, correct body posture and prevention of lymphedema; however, the PA level in women after BC treatment is unsatisfactory. In response to cancer treatment, some patients suffer from inactivity syndrome, e.g., in women during the first year after surgery for BC overall PA decreases to 11%, which is about two hours a week compared to the period prior to illness, and after additional radiotherapy and/or chemotherapy up to 50% [11,12].

PA is an important element of a healthy lifestyle, and it plays a major role in both primary and secondary prevention of lifestyle diseases. Recommended levels of PA after BC may increase survival by 20–50%, with strongest associations seen for recreational PA. Therefore, the promotion of PA to breast cancer survivors is paramount [13]. The most common form of PA in the daily life of every person is walking. Nordic walking (NW) training has become increasingly popular in recent years. This may be regarded as the most simple and accessible form of aerobic exercise. What is more, it can be easily adjusted to the age and exercise tolerance of the exercising person [14].

Existing scientific reports have confirmed that NW involves both the upper and lower extremities, leading to improvements in muscle strength and trunk muscle control and promoting an upright and balanced walking posture [14,15,16]. These factors may suggest a compensatory postural response (in the sagittal plane); however, only limited investigations have been conducted. Moreover, many authors have confirmed the positive effects of NW in rehabilitation [16,17,18]. In the case of BC survivors, a significant impact of this form of PA has been demonstrated with regard to improving the psychophysical condition [6,19]. NW has been shown to lead to a statistically significant increase in trunk muscle function compared to standard general gymnastics in previous studies [19], but these studies did not consider age as an important determinant of functional and postural changes in the trunk of women after BC treatment.

Optimal use of NW requires learning and applying the correct technique, which can be difficult for older people due to limited functional ability, prolonged stance phase [20] and shortened stride length [16] during the gait cycle. Therefore, it is believed that the effect of NW on changes in isokinetic endurance and spinal curvatures will be smaller in older women. The postural-functional condition deteriorates with age, which can be inhibited by properly selected forms of PA. The NW, on the basis of a review of the literature and previous studies, constitutes an appropriate form of activity for this purpose.

The aim of this study was to analyze the impact of NW on functional and postural changes of the trunk in women of different ages after BC.

## 2. Materials and Methods

The study was approved by the Local Bioethics Committee of the University of Physical Education in Wroclaw (8 March 2018). The trial was registered with the Australian and New Zealand Clinical Trials Registry (ANZCTR #: 12620000425998).

### 2.1. Study Group

We recruited women with BC to participate in an 8-week exercise program. To be eligible to participate in the study, women were approved by a physiotherapist under medical supervision based on the following inclusion criteria: diagnosis of BC (with radical or sparing surgery); completed adjuvant therapy in the form of radiotherapy, chemotherapy, or hormone therapy; female sex; age between 45 and 75 years; and at least 1 year after surgery. In the case of upper extremities lymphedema; or comorbidities (in the form of psychiatric, orthopedic, cardiac, neurological, or oncological diseases other than BC diseases), a medical certificate was required confirming that the patient’s psycho-physical condition allows safe participation in the study and exercise program.

Participants were recruited from self-help organizations such as Amazon Women’s Clubs in Kepno (Poland) through flyers, advertisements in local news outlets, and presentations in local oncology communities. Prior to the experiment, all participants were informed of the purpose of the experiment, how it would be conducted, and the possibility of resignation at any time during the research project. The participants gave written consent to participate in the research.

**Figure 1:**
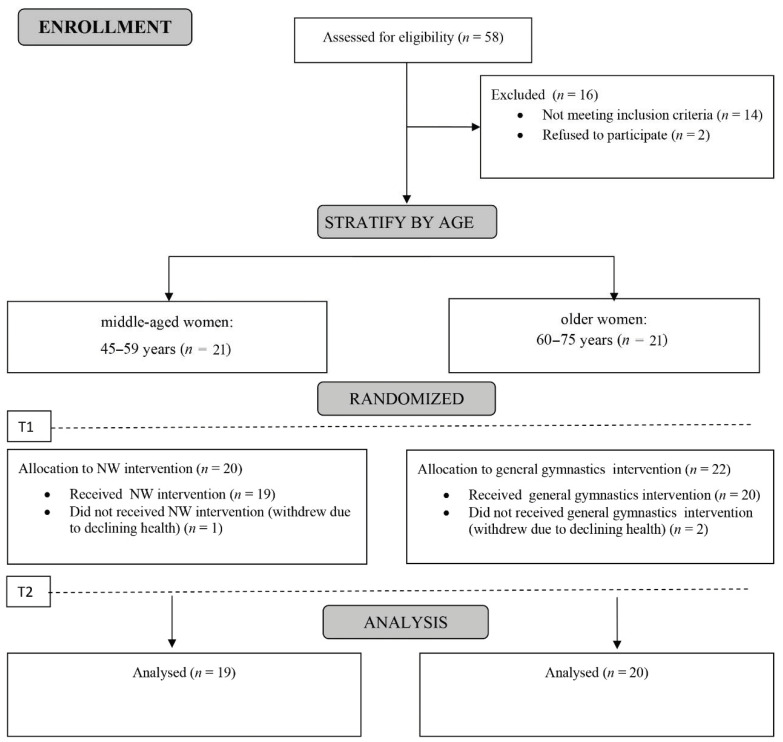
The participants flow diagram. Abbreviations T1: pre– and T2: post–training assessment of the sagittal spinal curvature parameters and isokinetic trunk muscle endurance.

A total of 58 patients were recruited for the study. Following inclusion and exclusion screening and confirmation for eligibility by a physician, 39 participants were included in the baseline sample (Figure 1).

### 2.2. Trunk Muscle Endurance Testing

The endurance of the trunk muscles was assessed using the Biodex Multi-Joint 3 Isokinetic Dynamometer. Measurements of alternating trunk flexion and extension (20 repetitions) with a maximum force in the shortest time at a selected angular velocity (120°/s) were recorded.

**Figure 2:**
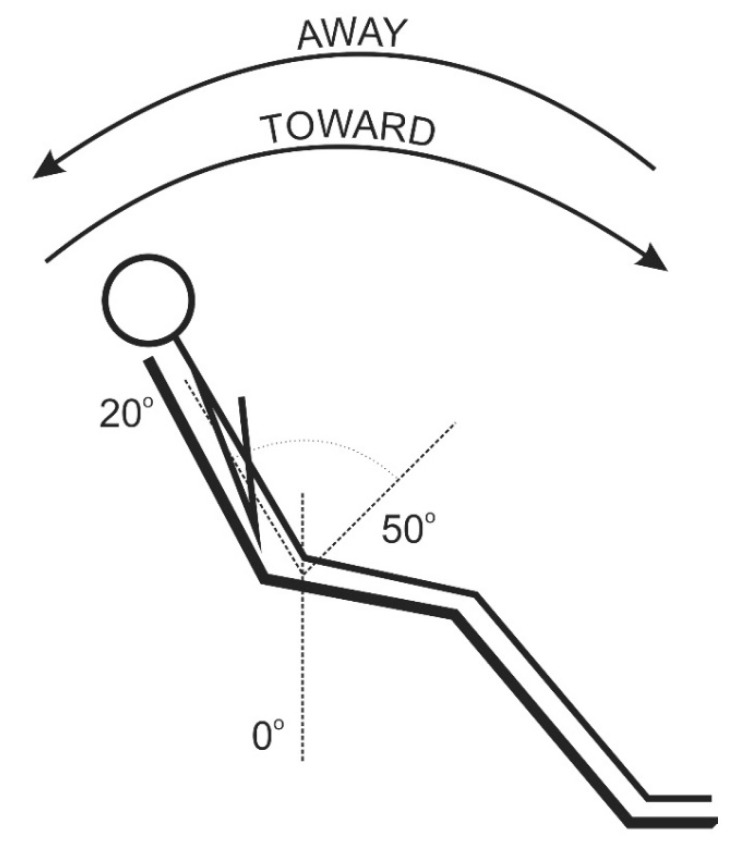
Isokinetic examination of the trunk muscles (semi-standing position).

The participant was placed in a semi-standing position [19,21,22,23,24] in a chair so that the arm of the dynamometer was positioned at the L5-S1 spine segment. The starting position began with a maximum extension of the trunk. In order for all participants to perform the same movement without pain, the range of motion was set at around 70° (extension 20°, flexion 50°). Additionally, to eliminate any supporting movements, the trunk and thighs were attached to the chair with straps (Figure 2). Each measurement was preceded by a verbal instruction and a warm-up exercise (several test runs).

The selection of total work—TW (J) and average power—AP (W) parameters for endurance measurements of the trunk muscles were based on existing studies using isokinetic tests [19,21,22,23,24]. TW represents the ability of the muscle’s capability to generate strength and maintain it throughout the range of motion. In turn, AP reflects the true measure of work rate intensity defined as TW divided by the time it takes to complete the work. TW and AP are determined earlier and faster than muscle strength (the ability to generate maximal muscle force). Therefore, these parameters are considered to be informative with respect to the functional performance preventing injuries [25,26].

### 2.3. Sagittal Spinal Curvatures Examination

The anterior and posterior curvatures of the spine were assessed using the fourth-generation Moiré apparatus (CQ Elektronik System, Wroclaw, Poland), which was based on direct observation of the participants using a camera. The first stage of the examination was to mark each participant’s spinous processes of the C7-S1 vertebrae along the spine. Next, the participants were recorded in a habitual standing position (with their back aligned toward the camera at a distance of 2.6 m) with their lower limbs upright in the knee joints, even distribution of body mass and feet parallel to each other. The participants looked straight ahead with their upper limbs hanging freely beside their trunk. Then, the participant’s spatial record based on the trunk image was entered into the data analysis software.

**Figure 3:**
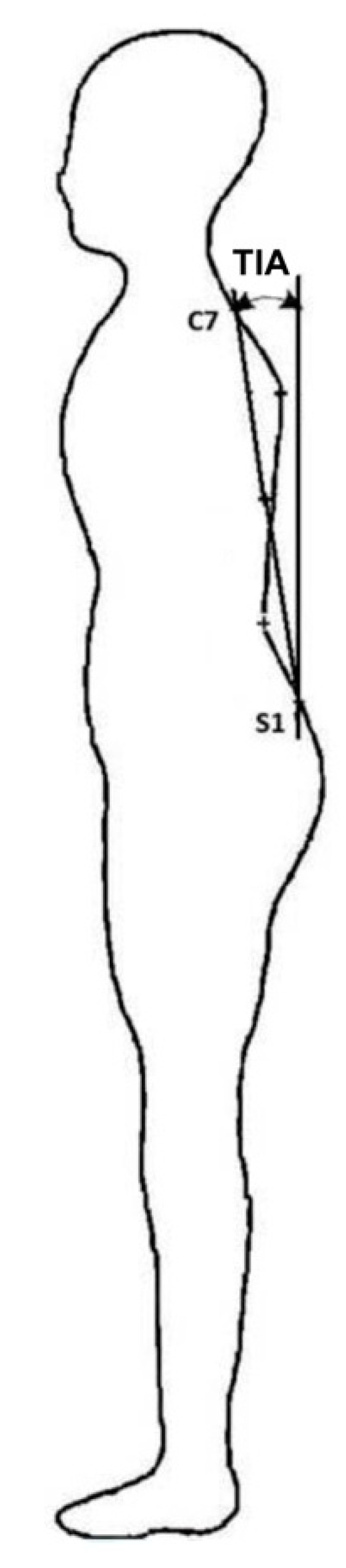
The trunk inclination angle (TIA). Abbreviations C7—seventh cervical vertebra, S1—first sacral vertebra.

The angular parameters of the spine, alpha angle (°)—the inclination of the lumbosacral section, beta angle (°)—the inclination of the thoracolumbar section, and gamma angle (°)—the inclination of the upper thoracic section, were selected for sagittal spinal curvature assessment [19,21,23,27]. Additionally, in order to determine the trunk inclination angle (TIA) in relation to the vertical and the trunk tilt by the sagittal degree between segments C7 and S1 and the vertical axis, data from the whole spine were calculated [28]. Negative angles indicate the forward inclination of the trunk relative to the vertical line (Figure 3).

### 2.4. Training Intervention

The NW and general gymnastics training intervention was continued for 8 weeks (16 training sessions) under the supervision of a NW instructor (SG) or a physiotherapist (CG). The programs consisted of two 45-min sessions per week (5-min warm-up, 35-min main part, and a 5-min cool-down). NW participants were familiarized with the NW technique: walking fluently, looking forward, holding the poles leaning back with elbows slightly flexed, extending the arms behind the trunk at the end of the pushing phase [29,30]. CG participants completed general gymnastics for women after BC treatment based on guidelines for cancer survivors [31] and as previously described (Table S1) [23]. Table 1 presents two forms of rehabilitation: NW as an innovative form of rehabilitation in the recovery process vs general gymnastics as a standard rehabilitation—widely offered by Amazon Associations in Poland.

For 45–50% of each exercise session, the training intensity was controlled at an endurance level of 65–70% of the participant’s maximum heart rate (HR max) based on their age and determined individually for each participant using a formula of 220–age [19,21,24,25]. In this way, a heart rate zone was designed individually for each woman, but the same HR max percentages were applied to the whole study group. The pulsometers (Polar RS 300 G1 model) automatically use vibration to control whether the effort is carried out in the desired heart rate zone. Moreover, during the rest period, patients monitored HR individually.

The intensity of the training intervention was increased by gradually decreasing the number of rest periods and increasing the distance covered (Table 2). Distance and number of the rest period were the average value for all Nordic walkers [19].

### 2.5. Statistical Analysis

The results of the study were analysed using Statistica 13.0 (StatSoft^®^, Cracow, Poland). The arithmetic mean and standard deviation were calculated as part of the basic descriptive characteristics for measurable data. The normal distribution of the parameters was examined for all groups using the Shapiro–Wilk test. No reasons were found to reject the hypothesis of a normal distribution. With regard to somatic data and the time since treatment, difference in mean values was evaluated using one-way analysis of variance (ANOVA). Analysis of variance (MANOVA) using the Wilks’ Lambda and least significant difference (LSD) post hoc tests was performed in a multivariate model with age distribution (middle-aged: 45–59 years and older: 60–75 years) to test whether NW training affects postural parameters and muscle function.

All values of tests were considered statistically significant at *p* < 0.05.

## 3. Results

The study group consisted of 39 BC survivors with a mean age, height and mass of 58.8 ± 7.30 years, 159.4 ± 5.84 cm and 74.4 ± 9.77 kg, respectively. Among them, 82% underwent radical mastectomy and 18% breast-conserving therapy. The cancer was more often located in the right breast (59%). The average time since surgical treatment was 6.13 ± 5.13 years. Additionally, 46% of the women underwent adjuvant therapy in the form of radiotherapy, 72% chemotherapy and 61% hormone therapy.

In terms of somatic characteristics, age subgroups were considered homogeneous. Detailed characteristics of the groups are presented in Table 3.

Based on the statistical analysis, a statistically significant main effect of training on isokinetic trunk muscle endurance and sagittal spinal curvatures in women after BC was achieved (F = 2.9, *p* = 0.02). The MANOVA revealed a significant group × time × age interaction for the TIA parameter (F = 4.9; *p* = 0.03). There was a significant negative tendency to increase forward inclination of the trunk (TIA parameter) in the group of older women after NW training.

Additionally, the post hoc analysis performed on the other postural parameters showed that the use of NW training in middle-aged women after BC treatment significantly reduces the size of thoracic kyphosis (gamma parameter).

In turn, post hoc testing of trunk strength and velocity parameters (TW and AP parameters) revealed changes in muscle function in response to NW training: in the middle-aged women group, a significant increase of trunk flexor and extensor muscle endurance was seen, while in older women, only a significant increase in flexor muscle endurance was seen.

No significant functional or postural changes were observed in CG in response to general gymnastics (Table 4 and Table 5).

## 4. Discussion

Effective exercise programs adapted to the needs, skills and psychophysical abilities of older people can slow the aging process in terms of muscle function [1]. This is especially important for oncological patients, including women after BC treatment, where functional changes resulting from treatment overlap with the aging process. The standard of conduct in this group are programs that aim to improve physical performance. General gymnastics is most commonly used in women after BC [32]. However, the ability to physically and independently perform daily activities remains a serious problem. The previous study [12] indicated that women after BC treatment have a low PA level, but this is not significantly different from healthy women.

NW is an increasingly popular and safe form of rehabilitation for the elderly, resulting in greater spinal stability through increased activation of the trunk muscles [29]. Spatiotemporal gait parameters change with age and as a result of external factors such as illness. After BC, women experience significant changes in the anatomical structure of the trunk, which consequently may cause postural defects but also result in a decrease in step length and walking velocity [33]. Moreover, it has been confirmed that weakness of the lower body muscles is associated with walking velocity in the elderly, while weakening of the lumbar region could lead to the development of bad posture [2,18]. Balzini et al. concluded that kyphotic posture is associated with slower gait and an increased support plane. These modifications can be attributed to more cautious behavior or as a compensatory mechanism to reduce stress on the painful spine. Furthermore, the increased forward inclination of the trunk impairs the use of normal postural compensatory strategies [2].

The intervertebral discs undergo the most dramatic age-related changes of all connective tissue, and can cause kyphosis [2]. Moreover, in the case of BC survivors, decreased muscle function, together with the adverse effects associated with treatment, can favor the occurrence of age-related kyphosis of the body posture [7,22,34]. However, it is possible to correct sagittal spinal curvatures by using effective compensatory motor strategies, even in the presence of severe misalignment [2].

Greater activity of several postural muscles during NW is possible due to arm swinging, required to keep the body in balance and to counterbalance the torque generated by the poles, which would otherwise rotate the upper trunk about the longitudinal axis of the body. NW is associated with longer strides, greater activation time of all trunk muscles and reduced co-activation of the spinal flexors and extensors. The trunk muscles are fundamental for the balance of the whole body, while co-activation of the neuromuscular system provides greater control of the trunk and the right spinal stability in different conditions [29].

NW may be used to improve the angular trunk balance, because it is a full-body exercise that strengthens the paraspinal muscles. In elderly women, the muscles around the spine were found to be aligned better after 12 weeks of NW training. Additionally, probably due to a simultaneous involvement and cooperation of the abdominal muscles, pelvic muscles and sensory nerve, the left–right balance was in equilibrium [17]. However, there are limited scientific reports examining the influence of NW on body posture.

In response to the NW intervention, we observed an adverse tendency to increase the forward inclination of the trunk in older BC survivors. Walking with poles requires the correct involvement of various body parts and coordination of movements. Learning the correct walking technique involves lengthening the stride, proper function of the upper limbs, placing the pole relative to the ground, and tilting the whole body forward. The inclusion of poles is a driving force behind a properly carried out NW technique; however, older people often incorrectly change the function of the poles and use them to increase support. When tilting the trunk forward during NW training, flexion of the hip and knee joints should be avoided; however, this is a characteristic standing position of the elderly due to their reduced posterior stability margin. The center of gravity of the body moves forward, which leads to trunk inclination in this direction and a subconscious attempt to balance the weight with supporting elements [27,29]. Additionally, Kocur and Wilk suggest that the correct functioning of the upper limbs is more difficult in older Nordic walkers, mainly in the last phase, which requires extension of the elbow and opening the hand with active muscle relaxation. This can result in a shortening of the stride, excessive effort and constant tension of the upper limb muscles [16]. As a consequence, these changes may result in rapid fatigue and adoption of a stooped posture characterized by excessive TIA.

A more stooped posture causes weakness and lengthening of the spinal extensor muscles, which play a key role in the pathophysiology of the flexed posture [2]. This translates into the results of the current study, as the significant increase in abdominal muscle function observed in the group of older BC survivors was accompanied by a lack of significant change in the trunk extensor group and a concomitant increase in the trunk inclination angle. However, in the younger group, a balanced significant increase in the activity of both muscle groups was observed with a concurrent reduction in the angle of thoracic spine kyphosis. Thus, NW training seems to be particularly beneficial for younger women after BC treatment.

We are aware of some limitations in our study such as a relatively small study group. It is possible that with a larger sample the results would be more reliable. Another major problem concerned the organization of the research design, where only self-referred motivated participants were recruited. Future research should be expanded to include the psychological condition, which was not presented as part of this study, but may possibly affect posture and muscle function and response to intervention. The study was organized and interpreted at the biomechanical level and needs to be extended to further studies with multidimensional assessment of the patient’s functional status. For this reason, the results can only be interpreted as biomechanical or physical changes and may not be related to patient’s pain, functional ability, or quality of life.

Regardless of the limitations, this research may serve as a valuable guidance to therapists and clinicians. Comparison of the results may be used to create suitable preventing programs aiming to correct age-specific sagittal spinal curvatures in women after BC treatment.

## 5. Conclusions

The use of NW training in middle-aged women after BC treatment is a safe and desirable form of rehabilitation for correction of body posture and trunk muscle endurance. In elderly women, due to an increase in TIA, indicating a stooped posture, attention should be paid to walking technique, proper use of poles and/or introduction of additional posture correction exercises. Further studies on a larger group are required to determine the definitive nature of the functional-postural changes in women after BC in response to the training interventions applied.

## Figures and Tables

**Table 1:** Description of exercise programs.

Session	Nordic Walking (NW)	General Gymnastics
Warm-up (5 min)	Aerobic activities, mobility exercises with breathing exercises	
Main part (35 min)	Learning and improving walking technique with the use of NW poles	General improving exercises using upper and lower limbs, back and abdominal strengthening exercises, balance and coordination exercises
Cool-down (5 min)	Stretching with breathing exercises	

**Table 2:** NW training sessions based on training distance, rest period and target heart rate zone.

Session	Distance (m)	Rest Period	Target HR Zone
1–4	6 × 400 = 2400	5 × 30 s	65–70% HR max
5–8	5 × 560 = 2800	4 × 30 s	
9–12	4 × 800 = 3200	3 × 30 s	
13–16	3 × 1200 = 3600	2 × 30 s	

Abbreviations: HR—Heart rate.

**Table 3:** Somatic parameters and the time since treatment in the study and control groups.

Somatic Parameters	Nordic Walking *n* = 19		General Gymnastics *n* = 20	
	<60 *n* = 11	>60 *n* = 8	<60 *n* = 9	>60 *n* = 11
Age (years)	53.09 ± 4.57	65.25 ± 2.82 *	52.7 ± 4.46	64.82 ± 4.28 *
Body height (cm)	161.27 ± 4.27	158.37 ± 7.96	162.00 ± 4.95	156.18 ± 5.06
Body mass (kg)	72.64 ± 10.10	77.62 ± 8.47	71.89 ± 4.46	75.91 ± 10.89
Time since surgery (years)	4.20 ± 3.99	8.75 ± 6.96	4.44 ± 3.64	7.54 ± 5.03

* Significant *p*-values (<0.05) based on the one-way ANOVA. <60 refers to middle-aged (45–59 years) and >60 to older participants (60–75 years).

**Table 4:** Pre- and post-training intervention changes in sagittal spinal curvatures parameters.

Sagittal Spinal Curvatures Parameters		Pre-	Post-	Within Group Change Scores	*p*-Value the LSD Post Hoc Test	*p*-Value Group × Time × Age Interaction (MANOVA)
		Intervention				
Alpha, lumbosacral angle (°)						
Nordic walking	<60	16.06 ± 4.50	13.99 ± 5.60	−2.07 (−4.10, −0.04)	0.17	0.77
	>60	16.75 ± 4.44	13.92 ± 5.36	−2.83 (−9.29, 3.64)	0.11	
General gymnastics	<60	16.53 ± 3.24	16.15 ± 5.16	−0.38 (−3.72, 2.96)	0.82	
	>60	15.64 ± 7.36	15.45 ± 7.10	−0.19 (−3.15, 2.76)	0.90	
Beta, thoracolumbar angle (°)						
Nordic walking	<60	13.16 ± 1.82	11.81 ± 1.44	−1.35 (−2.54, −0.17)	0.13	0.54
	>60	11.92 ± 6.97	9.94 ± 5.30	−1.98 (−5.09, 1.12)	0.06	
General gymnastics	<60	12.38 ± 3.81	11.54 ± 4.80	−0.84 (−3.09, 1.42)	0.40	
	>60	11.34 ± 4.58	11.04 ± 5.49	−0.30 (−2.50, 1.90)	0.74	
Gamma, upper thoracic angle (°)						
Nordic walking	<60	17.06 ± 3.48	14.77 ± 4.39	−2.29 (−4.54, −0.04)	<0.01 *	0.06
	>60	16.84 ± 2.58	17.01 ± 2.73	0.17 (−1.04, 1.39)	0.86	
General gymnastics	<60	16.31 ± 3.81	16.69 ± 4.14	0.38 (−1.04, 1.80)	0.49	
	>60	19.79 ± 3.35	19.23 ± 3.61	−0.56 (−2.72, 1.60)	0.68	
TIA, trunk inclination angle (°)						
Nordic walking	<60	−2.48 ± 2.50	−1.94 ± 2.63	0.54 (−0.21, 1.28)	0.25	0.03 *
	>60	−2.76 ± 3.34	−4.09 ± 2.63	−1.33 (−2.76, 0.11)	0.02 *	
General gymnastics	<60	−3.21 ± 3.90	−3.43 ± 4.17	−0.22 (−1.42, 0.97)	0.66	
	>60	−4.41 ± 2.91	−4.30 ± 3.53	0.11 (−1.04, 1.28)	0.80	

<60 refers to middle-aged (45–59 years) and >60 to older participants (60–75 years). Values are expressed as mean ± standard deviation for pre- and post-intervention data and as mean (95% confidence interval) for within group scores. LSD—least significant difference post hoc tests. * denotes statistical significance at *p* < 0.05.

**Table 5:** Pre- and post-training intervention changes in strength-velocity parameters of the trunk muscle.

Trunk Muscle Function		Pre-	Post-	Within Group Change Scores	*p*-Value the LSD Post Hoc Test	*p*-Value Group × Time × Age Interaction (MANOVA)
		Intervention				
Total work, trunk extensors (J)						
Nordic walking	<60	236.85 ± 216.23	510.73 ± 449.61	273.88 (116.85, 330.91)	<0.01 *	0.23
	>60	403.44 ± 353.80	545.32 ± 255.92	141,88 (−160.43, 344.18)	0.17	
General gymnastics	<60	211.64 ± 204.67	206.18 ± 183.06	−5.46 (−67.49, 56.55)	0.95	
	>60	168.25 ± 70.72	253.39 ± 200.92	85.14 (−82.01, 252.28)	0.33	
Total work, trunk flexors (J)						
Nordic walking	<60	247.52 ± 199.37	436.11 ± 309.96	188.59 (84.28, 292.90)	<0.01 *	0.63
	>60	273.38 ± 228.43	447.15 ± 159.75	173.77 (−40.46, 388.01)	<0.01 *	
General gymnastics	<60	197.78 ± 133.11	230.21 ± 118.90	32.43 (−31.47, 96.34)	0.55	
	>60	173.06 ± 80.81	240.67 ± 190.90	67.61 (−15.34, 150.55)	0.17	
Average power, trunk extensors (W)						
Nordic walking	<60	12.55 ± 11.72	30.09 ± 27.02	17.54 (6.36, 28.71)	<0.01 *	0.42
	>60	22.49 ± 20.99	33.81 ± 21.26	11.32 (−14.18, 36.83)	0.08	
General gymnastics	<60	11.18 ± 10.00	11.91 ± 10.22	0.73 (−4.44, 5.91)	0.90	
	>60	9.17 ± 4.58	13.04 ± 12.64	3.87 (−3.74, 11.48)	0.47	
Average power, trunk flexors (W)						
Nordic walking	<60	13.30 ± 9.70	23.35 ± 16.30	10.05 (4.52, 15.59)	<0.01 *	0.83
	>60	15.83 ± 13.93	27.95 ± 12.51	12.12 (−1.40, 25.63)	<0.01 *	
General gymnastics	<60	9.9 ± 7.43	12.30 ± 7.43	2.40 (−1.15, 5.84)	0.46	
	>60	9.89 ± 6.50	13.00 ± 11.34	3.11 (−1.54, 7.76)	0.28	

<60 refers to middle-aged (45–59 years) and >60 to older participants (60–75 years). Values are expressed as mean ± standard deviation for pre- and post-intervention data and as mean (95% confidence interval) for within group scores. LSD—least significant difference post hoc tests. * denotes statistical significance at *p* < 0.05.

## Data Availability

The trial was registered with the Australian and New Zealand Clinical Trials Registry (ANZCTR #: 12620000425998).

## Supplementary Materials

The following are available online at https://www.mdpi.com/1660-4601/18/5/2409/s1, Table S1: General gymnastics program.
