# Supplementary material for: The Influence of Nordic Walking on Isokinetic Trunk Muscle Endurance and Sagittal Spinal Curvatures in Women after Breast Cancer Treatment: Age-Specific Indicators
Source: Int J Environ Res Public Health. 2021 Mar 2;18(5):2409. doi: 10.3390/ijerph18052409 (PMC7967775; doi:10.3390/ijerph18052409)
Supplement: Supplementary file 1 [file ijerph-18-02409-s001.pdf]

Table S1. General gymnastics program

				Session			
Warm-up	Aerobic activities, mobility exercises and stretching with breathing exercise						
			Main muscle groups	1-4	5-8	9-12	13-16
General improving exercise	Mobility exercise	upper limb	scapular stabilizers, shoulder, elbow and wrist flexors and extensor	10 reps	12 reps	14 reps	16 reps
				x 5 sets			
		lower limb	hip and knee flexors and extensors, trunk stabilizers	10 reps	12 reps	14 reps	16 reps
				x 4 sets			
	Strenght exercise	back	low-back extensors and trunk stabilizers	10 reps	12 reps	14 reps	16 reps
				x 4 sets			
		abdomen	trunk flexors and low-back stabilizers	10 reps	12 reps	14 reps	16 reps
				x 4 sets			
	Balance exercise		trunk stabilizers	10 reps	12 reps	14 reps	16 reps
				x 2 sets			
	Coordination exercise			10 reps	12 reps	14 reps	16 reps
				x 2 sets			
	Forwards and backwards walking		hip and knee flexors and extensors, trunk stabilizers	2 reps	4 reps	6 reps	8 reps
				x 18 m			
Rest break	Relaxating with breathing exercises		inspiratory muscles	5 reps	4 reps	3 reps	2 reps
				30 sek			
Cool-down			Stretching with breathing exercise				
